# Assessment of Apparent Diffusion Coefficient Values as Predictor of Aggressiveness in Peripheral Zone Prostate Cancer: Comparison with Gleason Score

**DOI:** 10.1155/2014/263417

**Published:** 2014-02-09

**Authors:** Shayan Sirat Maheen Anwar, Zahid Anwar Khan, Rana Shoaib Hamid, Fahd Haroon, Raza Sayani, Madiha Beg, Yasir Jamil Khattak

**Affiliations:** ^1^Department of Radiology, The Aga Khan University Hospital, P.O. Box 3500, Stadium Road, Karachi 74800, Pakistan; ^2^Department of Medical Imaging, King Abdul Aziz Hospital, National Guards Health Affairs, P.O. Box 2477, Al-Ahsa 31982, Saudi Arabia; ^3^Radiology Department, Sultan Qaboos University Hospital, P.O. Box 38, 123 AL-Khod, Oman; ^4^Karachi X-Rays, U/S and CT Scan Centre, M. A. Jinnah Road, Karachi 296, Pakistan

## Abstract

*Purpose*. To determine association between apparent diffusion coefficient value on diffusion-weighted imaging and Gleason score in patients with prostate cancer. *Methods*. This retrospective case series was conducted at Radiology Department of Aga Khan University between June 2009 and June 2011. 28 patients with biopsy-proven prostate cancer were included who underwent ultrasound guided sextant prostate biopsy and MRI. MRI images were analyzed on diagnostic console and regions of interest were drawn. Data were entered and analyzed on SPSS 20.0. ADC values were compared with Gleason score using one-way ANOVA test. *Results*. In 28 patients, 168 quadrants were biopsied and 106 quadrants were positive for malignancy. 89 lesions with proven malignancy showed diffusion restriction. The mean ADC value for disease with a Gleason score of 6 was 935 mm^2^/s (SD = 248.4 mm^2^/s); Gleason score of 7 was 837 mm^2^/s (SD = 208.5 mm^2^/s); Gleason score of 8 was 614 mm^2^/s (SD = 108 mm^2^/s); and Gleason score of 9 was 571 mm^2^/s (SD = 82 mm^2^/s). Inverse relationship was observed between Gleason score and mean ADC values. *Conclusion*. DWI and specifically quantitative ADC values may help differentiate between low-risk (Gleason score, 6), intermediate-risk (Gleason score, 7), and high-risk (Gleason score 8 and 9) prostate cancers, indirectly determining the aggressiveness of the disease.

## 1. Introduction

Carcinoma of the prostate is a significant health issue affecting predominantly elderly men. In the year 2012 in United States of America (USA), estimated new cases and deaths from prostate cancer are 241,740 and 28,170 respectively [1]. Worldwide prostate cancer ranks third in cancer incidence and sixth in cancer mortality. The incidence of prostate cancer is not high in Pakistan, with an estimated figure of 3/100,000 of population. The possible explanation for this is lower life expectancy and no established screening programme for prostate cancer in Pakistan [2].

The diagnosis of prostate cancer is based on a digital rectal examination (DRE) and assessment of serum prostate specific antigen (PSA) followed by transrectal ultrasound (TRUS)-guided biopsy.

Magnetic resonance imaging (MRI) of prostate cancer with conventional T2-weighted imaging is routinely used for diagnosis and local staging of prostate cancer along with biopsy. The presence of extra capsular extension and seminal vesicle invasion are sought. However, the more recent application of functional MRI, including diffusion-weighted imaging (DWI), MR spectroscopy, and dynamic contrast enhanced MR, has strong potential to expand the role of MRI by noninvasive characterization of prostate cancer and providing more accurate information regarding tumor location, size, spread, and aggressiveness [3, 4].

Several studies have recently shown that DWI can help differentiate between benign and malignant prostatic tissue on the basis of lower apparent diffusion coefficient (ADC) values of prostate carcinoma in comparison with normal prostate tissue. The reported ADC values of prostate cancer in the peripheral zone range between 0.98 and 1.45 × 10^−3 ^mm^2^/s [3, 5–10].

The histopathology reference standard for measuring and reporting prostate cancer aggressiveness is the Gleason grading system. Gleason grades 1–5 correspond to progressively more poorly differentiated prostate cancer. A given tumor is assigned both a primary (most prevalent) and a secondary (second most prevalent) Gleason grade, and the sum of these grades yields the Gleason score (GS). Gleason scores are also used to describe tumors as low grade (Gleason score ≤ 6), intermediate grade (Gleason score 7), or high grade (Gleason score > 7) with respect to tumor aggressiveness [11].

The Gleason score decides the biological and prognostic behavior of the prostate tumor, achieved on transrectal biopsy or radical prostatectomy specimen. Accurate scoring is critical in decision of appropriate therapy in order to benefit the patient most, according to the risk stratification. For low-risk tumors (Gleason score < 7) no immediate treatment is required, that is, watchful waiting. For intermediate-risk (Gleason score = 7) monotherapy is offered and for high-risk prostate cancer (Gleason score > 7) combination therapy will be the best treatment option [12]. There is a dearth of scientific literature regarding utility of functional MRI in predicting tumor biology and behavior from south-east Asia. Therefore, we attempted to compare DWI and ADC values with Gleason score to determine the relationship between the two and explore new pathway for noninvasive assessment of tumor aggressiveness. Comparison of Mean ADC values with prior studies is given in Table 1.

## 2. Materials and Methods

### 2.1. Overview

A descriptive case series was conducted at the Radiology Department of Aga Khan University Hospital between June 2009 and June 2011. Data were retrospectively retrieved from the medical record system. The institutional ethical review committee granted exemption for patients' informed consent because data was retrospectively retrieved. As per departmental protocol, every contrast-enhanced study is performed after a written informed consent.

### 2.2. Study Population

Study sample consisted of 28 patients proved to have prostatic malignancy on basis of ultrasound guided sextant prostate biopsy at our department and later underwent MRI of pelvis for staging of disease. Patients were excluded if they had undergone prior surgery, radiotherapy, or hormonal therapy and contra-indications to MR imaging. In addition, patients were excluded if their biopsy was performed outside our institution. They were enrolled via non-probability purposive technique. Each patient underwent sextant biopsy and out of 28 patients total 168 biopsy samples were obtained. The 6 quadrants of prostate on MR imaging were taken as individual sample and analyzed.

### 2.3. Biopsy Technique

All biopsies were performed by credentialed radiologists using the same standard technique and 18-gauge core biopsy needles. Right and left half of each prostate gland was divided into a total of 6 zones (apex, mid, and base on each side). One core biopsy sample was obtained from each zone. Informed consent is taken by every patient in undergoing biopsy in our institution.

### 2.4. Imaging Protocol

All MRI scans were performed with 1.5 T machine (MagnetomAvanto, Siemens) using pelvic phased array coil. MRI pelvis protocol included sagittal, axial and coronal turbo spin echo T2-weighted images, coronal turbo spin echo T1-weighted images, axial turbo spin echo T1-weighted fat suppressed images, diffusion weighted axial images (*b*-value 50, 400, and 800 s/mm^2^) with ADC maps and post contrast fat suppressed sagittal, and coronal and axial T1-weighted images.

### 2.5. Image Analysis and Reader Procedure

Images were reviewed by 3 years' experienced radiologist trained in MRI and a senior resident on diagnostic workstation. All regions of interest (ROIs) were drawn on ADC maps with consensus and in case of difference in opinion; final judgment was taken from the third radiologist with 7 years' experience in MRI reporting. Assessment was based on 6 anatomical zones of prostate peripheral zone. The readers were aware of patient's prostatic malignancy but blinded to biopsy reports. Quantitative values of ADC were obtained from each quadrant by all three readers.

### 2.6. Data Analysis Plan

Data was entered and analyzed in SPSS 20.0 version. Proportions and mean ADC values of each Gleason score were calculated individually along with standard deviation. Range was also computed. Shapiro-Wilk test of normality was assessed as a numerical means for testing normality at *p* value > 0.05. A one-way between and within subjects analysis of variance (ANOVA) was conducted to compare the effect of ADC values on aggressiveness of tumor as predicted by Gleason score. *p* value < 0.05 will be taken as statistically significant. Tukey's post hoc analysis was computed to assess statistically significant difference among various levels of Gleason scores. (Such as low grade from intermediate and high grade and multiple comparisons). The 6 quadrants of each patient were analyzed individually and interrelated with Gleason scores derived from histopathological results.

## 3. Results

In our study, 28 men (mean age, 69.6 years ± SD 7.8; range, 53–88 years) underwent a total of 168 transrectal ultrasound-guided core biopsy of prostate. Sixty-three percent, that is, 107/168 of these sextant biopsies, were found to have prostate cancer. All Transrectal ultrasound-guided biopsies and sites of tumor were in the peripheral zone of the prostate gland. Twenty-eight tumors were identified in apex, 40 in mid zone, and 39 present in base. Twenty-six subjects (92%) had more than one quadrant involved by tumor on sextant biopsy and only two subjects had single site of involvement. In one patient, single quadrant from left lobe yielded tumor; however the sample was inadequate and Gleason score could not be calculated. The mean PSA level was 44.5 ng/mL ± SD, 78.5, range 1.1–395.6 ng/mL.

No special consideration was given to areas showing hemorrhage; however the number of quadrants involved was recorded. T2-weighted images for tumor assessment were not statistically evaluated.

### 3.1. MRI Imaging

The mean number of days between transrectal ultrasound-guided prostate biopsy and MRI was 14 ± SD 9 days, range 2–40 days. Ample number of biopsy-proven sites of prostate cancer was not detected on DWI. Eighty-nine (84%) biopsy-proven sites of prostate carcinoma were diffusion restricted. The remaining 17 (16%) biopsy-proven sites of cancer were not visible on DWI. The Gleason scores for the 89 quadrants visible and 17 quadrants not visible on DWI are given in (Table 2).

**Figure 1:**
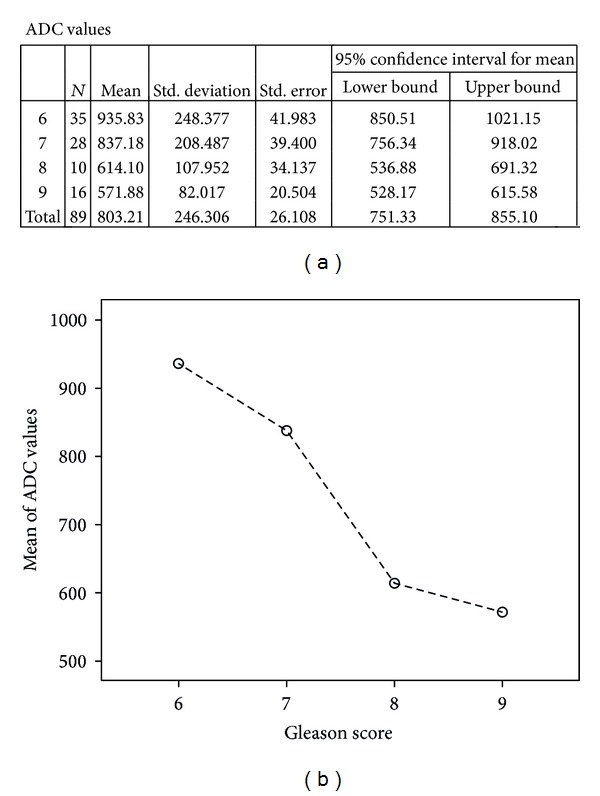
Means plot-inverse relationship between ADC values and Gleason score.

Total of 17 quadrants were not visible on DWI and these entire quadrants positive for tumor had Gleason scores of 6 and 7. Hence, we saw that the lower the Gleason score, the less densely packed the tumor and therefore less diffusion restriction of the water molecules. This hypothesis was supported by the means' plot showing inverse relationship between ADC values and tumor aggressiveness (Figure 1). Significant negative relationship was identified between ADC values in PZ cancer and tumor Gleason score.

**Figure 2:**
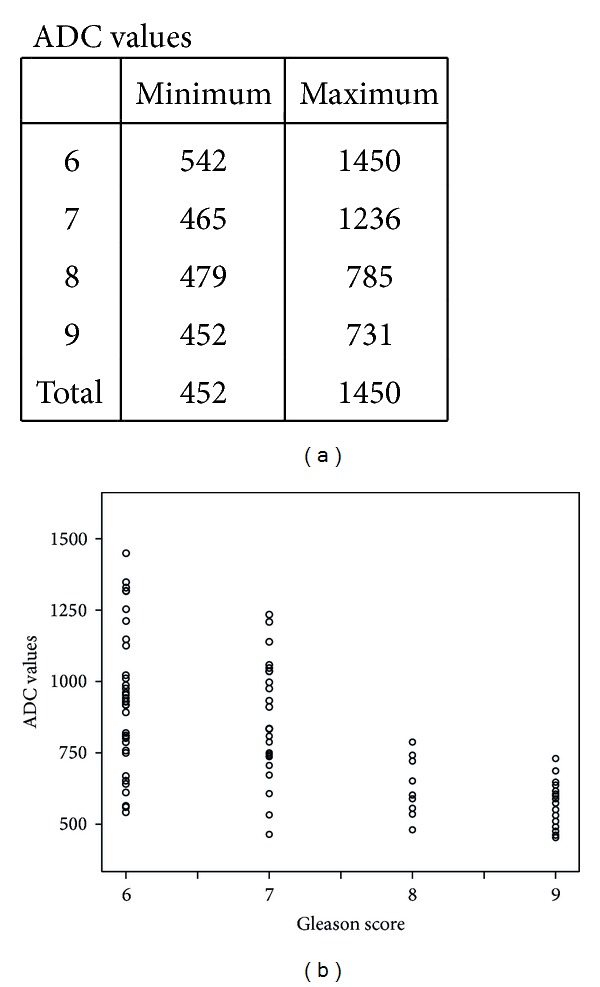
Scatter plot showing the relationship between ADC values in PZ cancers and Gleason scores.

For the 89 tumors visible on DWI, the mean ADC values ± SD were 803 ± 246 mm^2^/s, range: 452–1450 mm^2^/s. The radiologist observed variation and overlap in ADC value among the different groups (Figure 2). The mean ADC values of Gleason scores 6–9, visible on DWI are given in (Table 2).

In 56/168 (33.3%) quadrants proven to be benign prostatic tissue on biopsy had no diffusion restriction. Hemorrhage was observed in 10 quadrants which were of variable intensity on T1 and T2. The mean ADC value for the 56 cases of benign prostatic tissue was 1346 mm^2^/s ± 309 mm^2^/s, range: 2101–1011 mm^2^/s.

**Figure 3:**
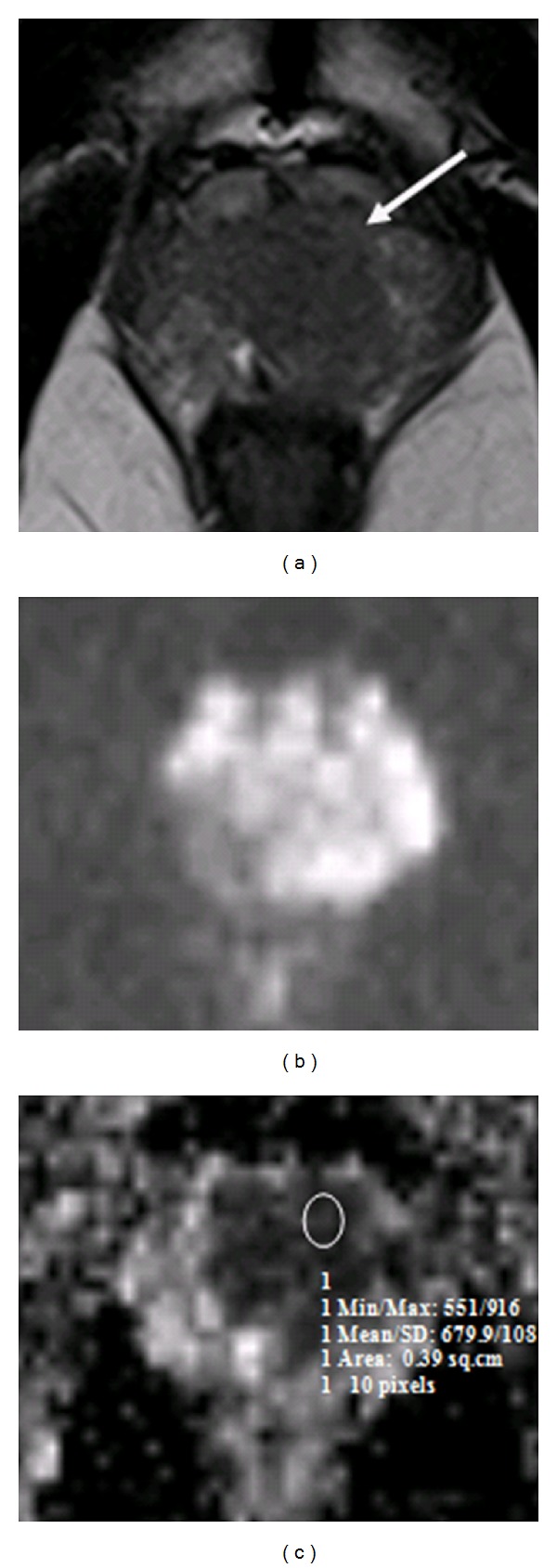
Patient with high-risk prostate cancer. Pelvic T2W transverse image (a) shows a well-defined low signal intensity region in the peripheral zone on the left base (arrow). (b) DWI; (c) an ROI drawn on ADC map shows restricted diffusion in corresponding left base PZ, scored as Gleason 9 on sextant biopsy.

**Figure 4:**
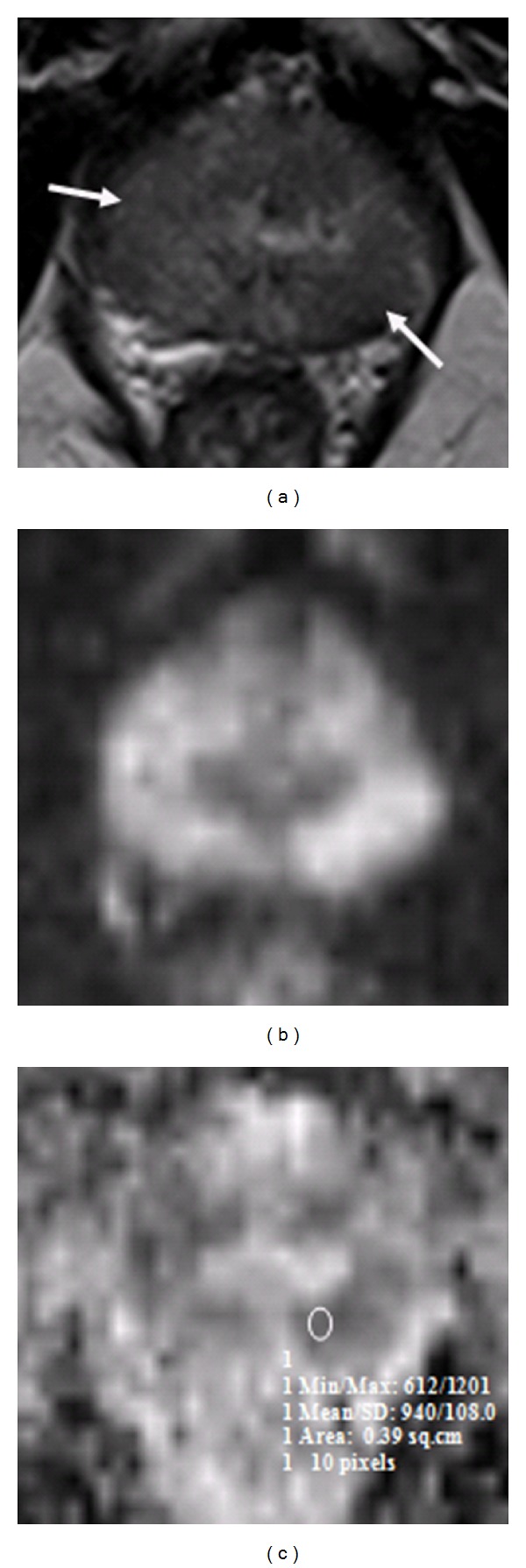
Patient with low-risk prostate cancer. Pelvic T2W transverse image (a) shows a well-defined low signal intensity region in the peripheral zone on the right and left mid zones (arrows). (b) DWI-hyper intense signal in corresponding areas. (c) A radiologist-determined region of interest on ADC map shows restricted diffusion in right and left peripheral zones, scored as Gleason 6 on sextant biopsy.

A one-way ANOVA was conducted between means of ADC values to compare relationship of ADC value with tumor aggressiveness, such as, low grade (Gleason score 6), intermediate grade (Gleason score 7), and high grade (Gleason scores 8 and 9) tumors. There was a highly significant effect of tumor aggressiveness on ADC values taken at *p* < 0.05 for various levels of Gleason scores. (*F*(3,85) = 15.2; *p* < 0.0001). Post hoc comparisons using Tukey's HSD test indicated that the mean ADC value for low grade tumors (*M* = 935.8 mm^2^/s, SD = 248.4 mm^2^/s) was significantly different than high grade tumors (*M* = 614.1 mm^2^/s, SD = 108 mm^2^/s) (*M* = 571.9 mm^2^/s, SD = 82 mm^2^/s). However, the intermediate grade tumor (*M* = 837.2 mm^2^/s, SD = 208.5 mm^2^/s) did not significantly differ from low grade but differed from high grade tumors. Taken together, these results suggest that tumor aggressiveness has strong inverse relationship with ADC value. Specifically, our results suggest that the more aggressive the tumor the lesser the ADC value. However, it should be noted that low grade and intermediate grade tumors have no statistically significant difference in ADC values. Imaging of two cases has been illustrated in the paper as Figures 3 and 4.

## 4. Discussion

Diffusion restriction in prostate cancer with corresponding signal drop outs on ADC mapping has been well documented in multiple prior studies [5, 6, 9, 16–19].

The pathophysiology behind these signals is increased water proton in rapidly growing tumor cells in extracellular as well as intracellular environment which have restricted movements and therefore give reduced ADC values compared to the normal healthy prostatic tissue [20–23].

Significant reduction in diffusion restriction and low ADC values in prostate cancer have been well established and few prior studies have studied the relationship between prostate cancer ADC value and aggressiveness. No such study has been performed in our population on this subject to date. Our study gives some interesting results which correlates with prior investigations.

In our observation of comparison between tumor aggressiveness and diffusion characteristics, we discovered inverse relationship between Gleason score and ADC values. There is an increase in Gleason score with falling ADC values. Considering tumor visibility on diffusion weighted images, their corresponding ADC values helped us to differentiate between low-risk (i.e., Gleason score 6) and high-risk (i.e., Gleason scores 8 or 9) prostate cancer (*p* < 0.0001) and between intermediate-risk (i.e., Gleason score 7) and high-risk (i.e., Gleason score 8 and 9) prostate cancer (*p* = 0.019 and *p* < 0.0001). Differentiation between low-risk (i.e., Gleason score 6) and intermediate-risk (i.e., Gleason score 7) prostate cancer was found insignificant (*p* = 0.226) which may be explained by the fact that there is less variability between their cell density and composition.

Arora et al. have concluded that prostate adenocarcinomas if multifocal have variable Gleason scores based on heterogeneity and this leads to difference between individual tumor focus Gleason score and overall Gleason score [24].

Similarly the cellular density is also variable with each focus and leads to overlap in the ADC value. Considering this overlap in the ADC value and retrospective nature of the study, we found that ADC value alone is not a good predictor of Gleason score and aggressiveness.

The inverse relationship between ADC values and cellular density is well established by Zelhof et al., suggestive of increasing diffusion restriction of water protons in adenocarcinoma with increasing cellular density and poor differentiation, hence indicating fall in ADC values with increasing tumor aggressiveness [7].

Earlier, Kim et al. [3] documented that ADC is a useful tool to differentiate between malignant and benign tissue in both peripheral and transitional zone based on 3 Tesla phased array coil study at 0 and 1000 s/mm^2^

*b* values. No correlation was established between ADC value and Gleason score of malignant tissue. Similar investigations were performed by Gibbs et al. [6] and Pickles et al. [5] at *b* values = 0 and 500 s/mm^2^, only differentiating tumor from normal peripheral zone.

Later, Yoshimitsu et al. [13] using phased array coil on 1.5 tesla machine and *b* values of 0, 500 and 1000 s/mm^2^ inculcated reverse association between tumor aggressiveness and ADC value keeping stepwise histopathology as gold standard on radical prostatectomy specimens. The parameters we deployed for our investigation and our results are somewhat similar to this study; however we used sextant biopsy histopathology as gold standard. Difference between well and poorly differentiating carcinoma was also significant (*p* = 0.019) similar to our result (*p* < 0.0001).

A recent report by Tamada et al. had similar results to our study. Their patient cohort of 125 healthy male volunteers and 90 prostate cancer patients was subjected to pelvic MRI using 1.5 Tesla machine, pelvic phased array coil, and maximum *b* value of 800 s/mm^2^. They found negative correlation between ADC value and Gleason score of cancer in peripheral zone proved on core biopsy specimen [8].

In our series of patients, 17 quadrants of biopsy were not diffusion restricted and 13 out of them had Gleason score of 6 (76.4%). This finding is explained by Langer et al., who inferred that sparse tumors had Gleason score of 6, having more water proton diffusibility and similar ADC values as adjacent normal peripheral tissue [25]. Tumor visibility and localization were therefore not improved by T2 combined with DWI and ADC maps, in cases of well-differentiated (low-risk) adenocarcinoma [14].

Our results were somewhat similar to Woodfield et al. [14] although we did not use endorectal coil and identical *b* values for ADC mapping. This infers that appropriate imaging technique and interpretation of images may give satisfactory results.

Another recent study by Yağci et al. [15] performed prospective study in 43 men with 1.5 Tesla MRI, using endorectal coil and maximum *b* value for DWI = 800 s/mm^2^. Yağci et al. found negative correlation between ADC value and histopathology results. They inferred that quantitative analysis of ADC value may help as a prognostic marker by indicating degree of tumor differentiation and aggressiveness. Our results affirm the above mentioned result. A number of limitations were observed in our study. First is the inclusion criterion of all biopsy proven cases leading to selection bias. Second limitation is keeping sextant core biopsy as gold standard rather than step-section histopathology of radical prostatectomy specimen. The false negative rate of standard sextant biopsy is 39% making it less accurate for diagnosis [26]. Secondly Gleason scoring achieved by transrectal sextant biopsy also undergrades the pathology compared to final pathological Gleason score of radical specimen and that too more for low-risk tumors [27].

Thirdly, standard sextant biopsy was used as gold standard to correlate Gleason score with quantitative ADC values on MR. Absolute matching of sextant maps of MRI and sextant biopsy samples is questionable. Though we only concentrated on peripheral zone, the sextant mapping of prostate on MRI and standard sextant biopsy was subjective and prone to error. Small tumors visible on imaging may be missed on biopsy sample giving discrepancy in results. We did not compare the results with final radical prostatectomy specimen Gleason scores as only 9/28 (32%) patients were subjected to radical prostatectomy in the study duration. Also, we used phased array body coil. An endorectal coil was not employed in this study due to nonavailability in our institution and secondly it is often not well tolerated. It also increases susceptibility effects [28]. Central and transitional zones were not assessed and interobserver variability was not evaluated. Lastly, there is no general agreement on the optimal *b* value for DWI of the prostate. The employment of higher MR field strengths and higher *b* values can refine tumor detection.

In conclusion, ADC values of prostate carcinoma in peripheral zone on pelvic MRI performed at 1.5 Tesla, using phased array body coil and *b* values of 50, 400, and 800 s/mm^2^, may help to assess aggressiveness of tumor and may help differentiate between low-risk (Gleason score = 6) and high-risk (Gleason score = 8 or 9) and intermediate-risk (Gleason score = 7) and high-risk (Gleason score = 8 or 9) prostate cancer. It may identify patients at higher risk of recurrence and bad prognosis and help direct their appropriate treatment plan. Prospective study with larger sample size, *b* value of 1000 s/mm^2^ and keeping radical prostatectomy specimen as gold standard, needs to be conducted in the future.

## Figures and Tables

**Table 1:** Comparison of ADC values with previous studies.

	Low-risk adenocarcinoma mean ADC mm^2^/s	Moderate risk adenocarcinoma mean ADC mm^2^/s	High-risk adenocarcinoma mean ADC mm^2^/s
Yoshimitsu et al. [13]	1.19 × 10^−3^	1.10 × 10^−3^	0.93 × 10^−3^
Woodfield et al. [14]	0.86 × 10^−3^	0.702 × 10^−3^	0.68 × 10^−3^
Ya$\overset{ˇ}{\text{g}}$ci et al. [15]	1.18 × 10^−3^	1.05 × 10^−3^	0.84 × 10^−3^
Shayan et al.^a^	0.93 × 10^−3^	0.83 × 10^−3^	0.57 × 10^−3^

^a^Current study results.

**Table 2:** Number of diffusion positive and negative quadrants according to the sextant based prostate biopsy results.

Gleason scores	Diffusion positive quadrants	Diffusion negative quadrants
6	35	13
7	28	4
8	10	0
9	16	0

Total	89	17
